# Prognostic Value of Survivin in Patients with Non-Small Cell Lung Carcinoma: A Systematic Review with Meta-Analysis

**DOI:** 10.1371/journal.pone.0034100

**Published:** 2012-03-23

**Authors:** Lou Qian Zhang, Jun Wang, Feng Jiang, Lin Xu, Fu Yin Liu, Rong Yin

**Affiliations:** 1 Department of Thoracic Surgery, Jiangsu Cancer Hospital, Affiliated Cancer Hospital of Nanjing Medical University, Nanjing, China; 2 Department of Chemotherapy, Jiangsu Province Geriatric Institute, Jiangsu Province Official Hospital, Nanjing, China; Univesity of Texas Southwestern Medical Center at Dallas, United States of America

## Abstract

**Purpose:**

The potential prognostic value of survivin in resected non-small cell lung carcinoma (NSCLC) is variably reported. The objective of this study was to conduct a systematic review of literatures evaluating survivin expression in resected NSCLC as a prognostic indicator.

**Methods:**

Relevant literatures were identified using PubMed, EMBASE and Chinese Biomedicine Databases. We present the results of a meta-analysis of the association between survivin expression and overall survival (OS) in NSCLC patients. Studies were pooled and summary hazard ratios (HR) were calculated. Subgroup analyses and publication bias were also conducted.

**Results:**

We performed a final analysis of 2703 patients from 28 evaluable studies. Combined HRs suggested that survivin overexpression had an unfavorable impact on NSCLC patients' survival with no evidence of any significant publication bias (HR = 2.03, 95%CI: 1.78–2.33, Egger's test, P = 0.24) and no severe heterogeneity between studies (I^2^ = 26.9%). Its effect also appeared significant when stratified according to the studies categorized by histological type, HR estimate, patient race, cutoff point (5%, 10%), detection methods and literature written language except for disease stage. Survivin was identified as a prognostic marker of advanced-stage NSCLC (HR = 1.93, 95%CI: 1.49-2.51), but not early-stage NSCLC (HR = 1.97, 95%CI: 0.76-5.14), in spite of the combined data being relatively small.

**Conclusion:**

This study shows that survivin expression appears to be a pejorative prognostic factor in terms of overall survival in surgically treated NSCLC. Large prospective studies are now needed to confirm the clinical utility of survivin as an independent prognostic marker.

## Introduction

Lung cancer is the leading cause of death from cancer around the world, accounted for an estimated 157,300 deaths in the United States in 2010 [1]. Non–small cell lung carcinoma (NSCLC) accounted for approximately 85% of the cases [2]. Despite recent advances made in clinical and experimental oncology, the prognosis of lung cancer is still unfavorable, with a 5-year overall survival rate of only around 11% [3]. Several independent prognostic factors for survival in NSCLC patients have been identified: performance status, disease stage, age, sex, and amount of weight lost [4]. The most important prognostic factor for survival is tumor stage, primarily because early stage disease is amenable to completely surgical resection, hopefully before the tumor cells have acquired the ability to metastasize. However, even in the early stage of the disease, about 30% of patients suffer from relapse and die within 5 years of surgery [5]. Although these prognostic parameters do reflect biological features of both tumor and patient, they do not allow adequate prediction of outcome for the individual patient. The discovery of molecular biological prognostic factors should aid in a more accurate prediction of clinical outcome and may also reveal novel predictive factors and therapeutic targets [6]. Hundreds of studies have evaluated prognostic markers that have an association with some clinical outcome, typically overall or recurrence-free survival in lung cancer. Of these, the three important pathways in lung cancer: cell cycle regulation, apoptosis, and angiogenesis are widely investigated.

Survivin also called baculoviral inhibitor of apoptosis repeat-containing 5 (BIRC5) is a member of the inhibitor of apoptosis (IAP) family, which is one of the most cancer-specific proteins identified to date, being unregulated in almost all human tumors. Biologically, survivin has been shown to inhibit apoptosis, enhance proliferation and promote angiogenesis [7]–[9], which is expressed highly in most human tumors and fetal tissue, but is undetectable in most terminally differentiated cells [10]. Because of larger difference in expression between normal and malignant tissue and its causal role in cancer development, survivin is currently attracting considerable attention as a cancer prognostic indicator and a new target for anti-cancer therapies. Strategies under investigation to target survivin include antisense oligonucleotides, siRNA, ribozymes, immunotherapy and small molecular weight molecules (for review, see Refs.[11]). The translation of these findings to the clinic is currently ongoing with a number of phase I/II clinical trials targeting survivin in progress.

The expression of survivin has been reported to be a promising prognostic indicator, associated with a worse overall survival. However, evidence regarding the prognostic value of survivin with respect to overall survival in NSCLC remains controversial. In order to clarify this question, we performed this systematic review of the literatures with methodological assessment and meta-analysis.

## Results

### Literature Selection and Characteristics

A total of 317 potentially relevant citations were retrieved after initial databases search. The title and abstract of relevant articles were read by two authors independently. Two hundred and seventy-three citations were excluded from analysis after the first screening based on abstracts or titles, leaving 44 available for further full text review. After carefully reading the full text articles, 6 were excluded because they were reviews instead of observational studies. Five were excluded because they investigated the correlation with clinicopathological variables not survivals. Meanwhile, another 4 studies were excluded due to lacking of sufficient survival data. Additionally, 2 studies were found by hand search of the reference lists. As a result, 31 eligible studies including 2984 NSCLC cases were included in this meta-analysis [12]–[42]. The basic feature descriptions of the 31 studies are summarized in Table 1. Briefly, study sample sizes ranged from 43 to 219, 23 studies were conducted in Asian populations, while others were European populations. Twenty-five studies reported two or more subtypes of NSCLC, while 4 were about adenocarcinoma only. Fifteen studies were of I–III stage and 7 were of all stages. Most of the studies investigated survivin by IHC (24 studies), 6 studies using reverse transcription-polymerase chain reaction (RT-PCR), only one study identified survivin by fluorescence in situ hybridization (FISH) [38]. In addition, all the studies investigated survivin expression using lung cancer tissues except one study which detected the expression with circulating cancer cells [23].Twenty-four studies reported survivin as an indicator of poor prognosis, while the other seven studies showed no significant impact on overall survival.

**Table 1:** Characteristics and results of eligible prognostic studies evaluating surviving.

First Author	Year	Source of Patients	N. of Patient	Histology	Method	Stage	N. of Positive	Cutoff value	HR Estimate	HR	95%CIs
Kim [12]	2011	South Korea	151	SCC	TMA	I–IV	116	5%	HR	2.05	1.15–3.58
			93	ADC	TMA	I–IV	62	5%	HR	4.51	1.71–11.93
Zhang Q [13]	2010	China	74	ADC	IHC	I–III	25	10%	HR	2.16	1.37–3.75
Zhang JR [14]	2010	China	60	SCC&ADC	IHC	I–III	56	5%	HR	2.94	1.89–4.57
Yang [15]	2010	China	60	SCC&ADC	TMA	I–IV	31	5%	Sur. Curve	1.86	1.09–3.78
Weng [16]	2010	China	50	NSCLC	IHC	I–III	39	NA	HR	5.22	1.20–22.61
Porebska [17]	2010	Poland	74	NSCLC	IHC	I–IV	39	20%	Sur. Curve	0.98	0.28–3.44
Nakashima [18]	2010	Japan	122	NSCLC	IHC	I–IIIB	64	25%	HR	2.13	1.49–3.03
Lv [19]	2010	China	70	SCC&ADC	IHC	I–III	52	30%	HR	4.02	1.73–9.39
Grossi [20]	2010	USA	87	NSCLC	TMA	IIIA–N2	62	50%	HR	1.61	0.94–2.77
Dai [21]	2010	China	66	NSCLC	RT-PCR	IB–IIIA	33	0.413	HR	1.493	1.12–2.13
Chen [22]	2010	China	72	NSCLC	RT-PCR	IIIB–IV	36	0.467	HR	2.12	1.22–3.11
Yie [23]	2009	China	78	SCC&ADC	RT-PCR	I–IV	33	1.02	HR	1.51	1.06–3.63
Yamashita [24]	2009	Japan	47	NSCLC	RT-PCR	I–III	28	NA	HR	0.62	0.22–1.75
Mohamed [25]	2009	Japan	78	NSCLC	IHC	IIIA–N2	68	10%	HR	2.21	1.26–3.89
Hoshil [26]	2009	Japan	100	SCC&ADC	IHC	I–IIIB	76	10%	HR	1.73	1.04–2.97
Chen [27]	2009	China	80	ADC	IHC	III–IV	41	10%	Sur. Curve	1.81	1.05–3.13
Li [28]	2008	China	91	SCC&ADC	IHC	I–III	46	10%	HR	1.87	1.04–3.34
Bria [29]	2008	Italy	116	NSCLC	IHC	I–IIIA	82	20%	HR	1.83	1.01–3.30
Zhu [30]	2007	China	213	NSCLC	IHC	I–II	43	10%	HR	0.80	0.41–1.55
Yoo [31]	2007	Korea	219	NSCLC	IHC	I–IIIA	6	10%	HR	1.12	0.35–3.54
Wang [32]	2006	China	115	NSCLC	IHC	I–II	72	5%	HR	3.73	1.66–8.39
Vischioni [33]	2006	Netherlands	138	NSCLC	IHC	I–IIIA	127	5%	Logrank +p	0.78	0.49–1.26
Atikcan [34]	2006	Turkey	58	SCC&ADC	IHC	I–IIIA	28	25%	HR	3.73	1.53–9.05
Akyurek [35]	2006	Turkey	78	NSCLC	IHC	I–IV	50	10%	Sur. Curve	2.28	1.17–4.43
Zhou [36]	2005	China	43	SCC&ADC	IHC	I–III	34	5%	Sur. Curve	3.14	1.27–9.78
Shinohara [37]	2005	USA	144	NSCLC	IHC	I–II	105	25%	HR	2.74	1.29–5.79
Karczmarek [38]	2005	Poland	60	NSCLC	FISH	IIB–III	35	NA	HR	4.27	3.51–5.03
Kren [39]	2004	USA	102	SCC&ADC	IHC	I–IIIA	54	15%	Sur. Curve	2.16	1.34–3.44
Falleni [40]	2003	Italy	83	NSCLC	RT-PCR	I	44	25n	Sur. Curve	0.86	0.53–1.37
Ikehara [41]	2002	Japan	79	ADC	IHC	I–IV	41	10%	Sur. Curve	4.16	1.60–10.30
Monzo [42]	1999	Spain	83	NSCLC	RT-PCR	I–IIIA	71	NA	HR	2.20	1.10–4.50

N., number; ADC, adenocarcinoma; SCC, squamous cell carcinoma; NSCLC, non-small cell lung cancer; IHC, immunohistochemistry; TMA, tissue microarray; FISH, fluorescence in situ hybridization; RT-PCR, reverse transcription-polymerase chain reaction; NA, not applicable; HR, hazard ratio; Sur. Curve, survival curve.

### Quality Assessment

Overall, the global quality score of the included studies ranged from 44.6 to 62.0% with a mean of 55.2% (as Table 2 shows). Concerning the global score, there was no statistically significant difference between the 24 positive and the 7 negative trials (mean of 54.4% versus 55.2%, p = 0.67). There was no statistical difference in global score between studies that performed on Asian (n = 22) or non-Asian populations (n = 9), with scores of 51.6% and 54.5%, respectively (p = 0.25). Moreover, there was no significant disparities for the effective value of overall survival was determined (HR: 54.0%, Sur. Curve: 52.6%, p = 0.39).The absence of a significant quality difference between significant and non-significant studies made it possible to perform this quantitative aggregation of the survival results.

**Table 2:** Results of the methodological assessment by the European Lung Cancer Working Party score.

	Number of studies	Global Score (%)	Design(/10)	Laboratory methodology(/10)	Generalizability(/10)	Results analysis(/10)
All studies	31	55.2	5.4	5.7	5.3	5.2
Positive	24	54.4	5.3	5.4	5.5	5.0
Negative	7	55.2	5.4	6.1	5.2	5.4
*p*		0.67	0.76	0.42	0.12	0.66
Asian	22	51.6	4.9	4.8	5.0	4.9
Non-Asian	9	54.5	5.6	5.3	5.2	5.5
*p*		0.25	0.09	0.32	0.26	0.34
HR	22	54.0	5.6	5.6	5.4	5.3
Sur. curve	8	52.6	4.8	5.3	5.2	4.8
*p*		0.39	0.028	0.65	0.14	0.08

Score distributions are summarized by the median values; Negative, no significant prognostic factor for survival; Positive, as significant poor prognostic factor for survival; HR, Hazard ratio.

### Assessment of heterogeneity and meta-analysis

Highly significant heterogeneity was detected when all studies were pooled (chi-squared = 110.45, I^2^ = 71.9%, p<0.001), then the source of heterogeneity was explored using meta-regression analysis. One study investigated survivin expression by FISH [38], one study failed to report time-to-event data directly [33] and one study investigated survivin in only stage I NSCLC [40] were the main source of heterogeneity. After excluding them, the heterogeneity dropped sharply with no significant changes to the summary HR (chi-squared = 38.32, I^2^ = 26.9%, p = 0.092). In order to make a conservative estimate, random-effects meta-analyses were used to account for interstudy heterogeneity and to summarize the prognostic value of survivin across studies.

After excluding the 3 studies, the meta-analysis was performed on the 28 remaining studies. The main results of this meta-analysis are presented in Table 3. Overall, the pooled HR for all evaluable studies evaluated survivin expression in NSCLC was 2.03 (95%CI: 1.78–2.33). No individual studies influence the summary HR found by one-way sensitivity analysis, indicating that survivin overexpression was an indicator of poor prognosis for NSCLC patients.

**Table 3:** Summarized HRs of overall and subgroup analyses for survivin on NSCLC survival.

	N. of studies	Number of patients	Random effects HR(95%CIs)	Heterogeneity test		
				chi-squared	I^2^	P-value
Overall	28	2703	**2.03 (1.78–2.33)**	38.32	26.9%	0.092
Written Language						
English written	19	1929	**1.93 (1.69–2.21)**	20.18	5.8%	0.384
Non English written	9	776	**2.31 (1.66–3.21)**	16.57	51.7%	0.035
HR Estimate						
HR	21	2207	**2.01 (1.71–2.37)**	33.49	37.3%	0.041
Sur. Curve	7	516	**2.13 (1.65–2.74)**	4.58	0.00%	0.599
Histological type						
ADC	4	326	**2.50 (1.67–3.73)**	4.16	27.9%	0.247
ADC&SCC	9	662	**2.24 (1.83–2.75)**	8.24	2.90%	0.410
NSCLC	15	1565	**1.82 (1.48–2.23)**	21.84	35.9%	0.082
Methods						
IHC	23	2297	**2.16 (1.87–2.49)**	28.06	18.0%	0.214
RT-PCR	5	346	**1.62 (1.21–2.16)**	5.59	28.5%	0.232
Ethnicity						
Asian	22	2097	**2.07 (1.75–2.44)**	35.51	38.1%	0.034
Non-Asian	6	606	**1.95 (1.51–2.53)**	2.76	0.00%	0.736
Tumor stage						
I–II	3	472	1.97 (0.76–5.14)	10.08	80.2%	0.006
I–III	15	1301	**2.07 (1.72–2.48)**	19.73	29.0%	0.139
I–IV	6	613	**2.13 (1.57–2.89)**	7.18	16.5%	0.304
III–IV	4	317	**1.93 (1.49–2.51)**	0.86	0.00%	0.836
Cutoff value						
5%	6	522	**2.66 (2.05–3.47)**	4.17	0.00%	0.525
10%	9	1012	**1.84 (1.44–2.36)**	11.01	27.3%	0.201

**Figure 1:**
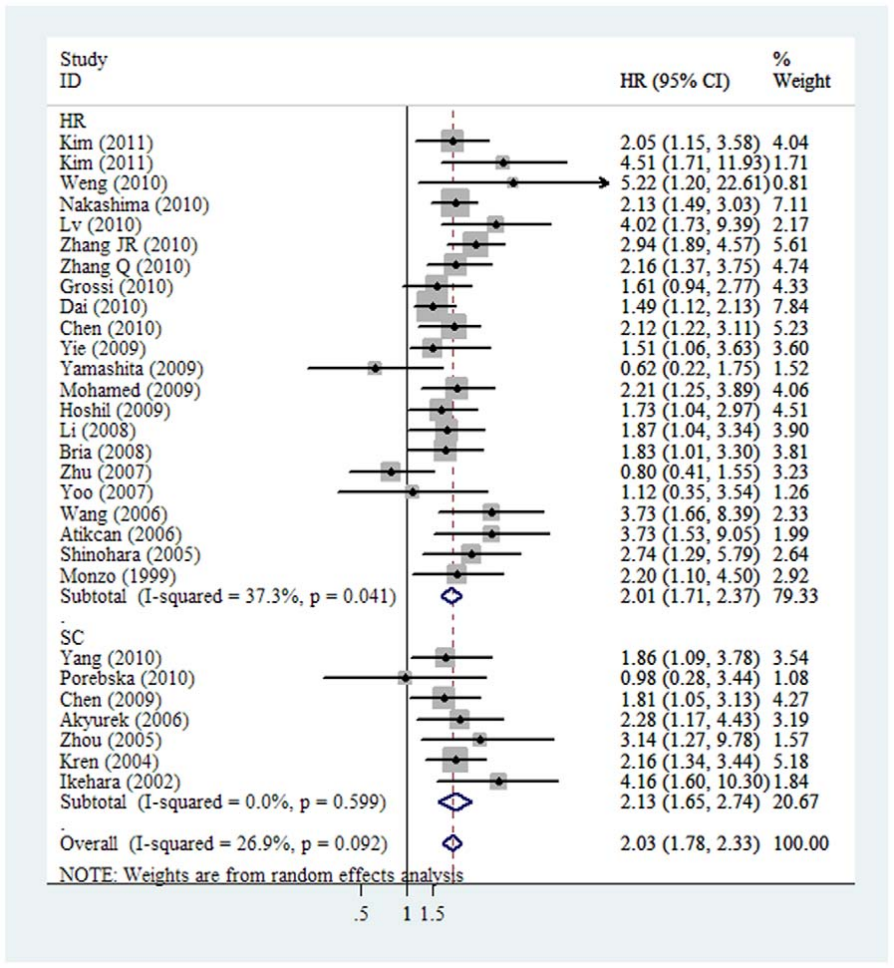
The association between survivin overexpression and overall survival of NSCLC stratified by tumor stage. The summary HR and 95% CIs were shown (according to the random effect estimations).

**Figure 2:**
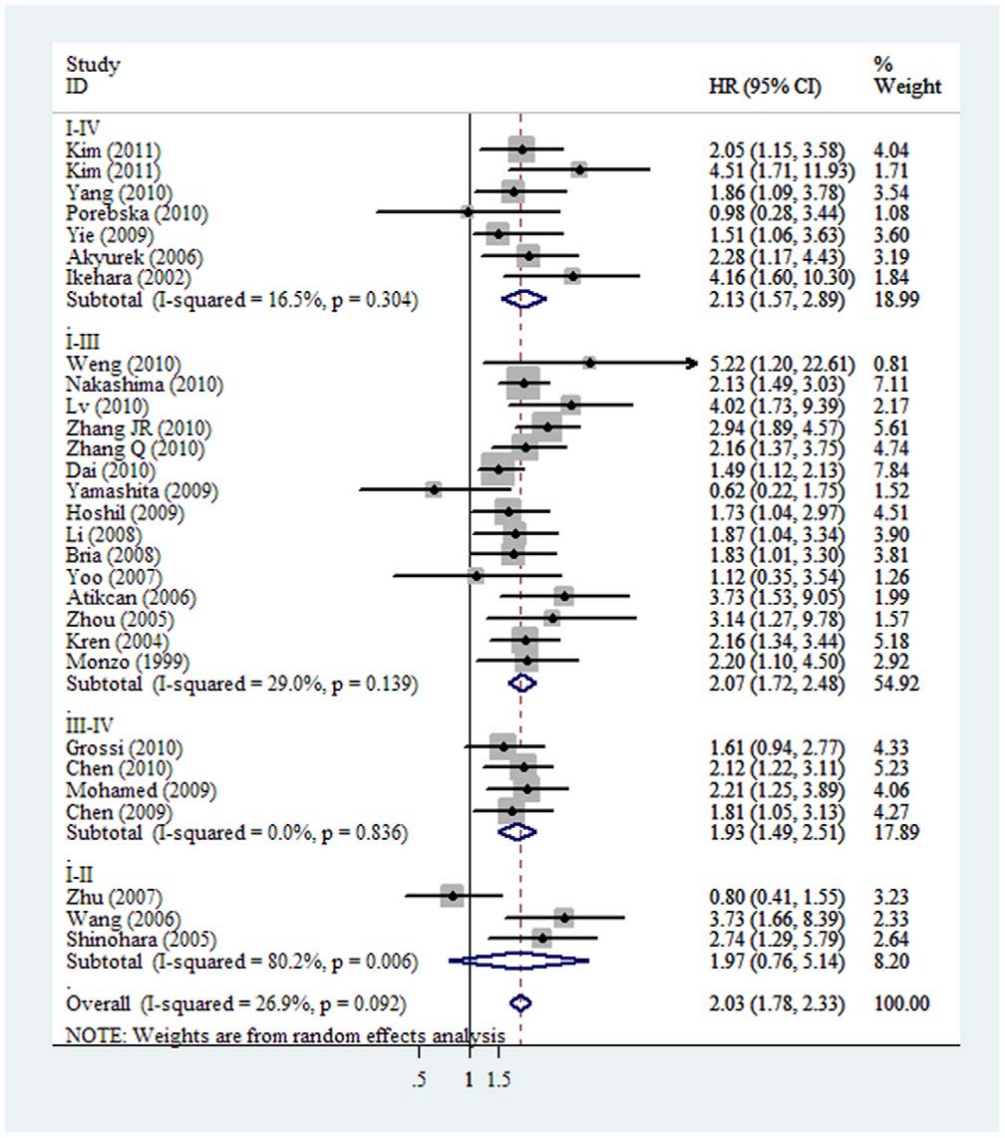
The association between survivin overexpression and overall survival of NSCLC stratified by HR estimation. The summary HR and 95% CIs were shown (according to the random effect estimations).

N., number; HR, hazard ratio; NSCLC, non-small cell lung cancer. ADC, adenocarcinoma; SCC, squamous cell carcinoma;

When grouped according to the ethnicity, the combined HRs of Asian studies and non-Asian studies were 2.07 (95%CI: 1.75–2.44) and 1.95 (95% CI: 1.51–2.53), respectively. In the subgroup analysis according to the method of survivin detection used, the combined HR was 2.16 (95%CI: 1.87–2.49) for IHC and 1.62 (95%CI: 1.21–2.16) for RT-PCR. When stratified according to literature written language, the combined HRs of both English and non-English literatures showed an inverse effects on survival (HR = 1.93 and 2.31, separately). Although we also observed statistically significant effects of survivin expression on survival from studies reported all stages with an HR of 2.13 (95% CI: 1.57–2.89) and from 15 studies reported stage I–III with an HR of 2.07 (95% CI: 1.72–2.48), when we aggregated the studies that reported results for early-stage and advanced-stage NSCLC, the combined HR were 1.97 (95% CI: 0.76–5.14) and 1.93 (95% CI:1.49–2.51) with 3 and 4 studies in each arms, respectively (Figure 1). Furthermore, we aggregated the studies separately according to histological subgroups, the summary HR of the studies investigated NSCLC as a whole was 1.82 (95% CI: 1.48–2.23). The combined HRs were 2.50 (95% CI: 1.67–3.73) and 2.24 (95% CI: 1.83–2.75) based on four studies of adenocarcinoma and nine of squamous cell carcinoma and adenocarcinoma, respectively. When the HRs derived from Cox regression analysis of the 21 evaluable studies were aggregated, the combined HR was 2.01 (95% CI: 1.71–2.37). It indicated that survivin was an independent prognostic marker in resected NSCLC. Moreover, when the survival data calculated indirectly from Kaplan-Meier based survival curve were pooled, the combined HR was 2.13 (95% CI: 1.65–2.74), also suggesting survivin status was of prognostic value (Figure 2). Finally, grouped according to the positive threshold for survivin expression, as defined by the studies' authors, the combined HRs of 5% and 10% cutoff value were 2.66 (95% CI: 2.05–3.47) and 1.84 (95% CI: 1.44–2.36), separately.

### Publication bias

**Figure 3:**
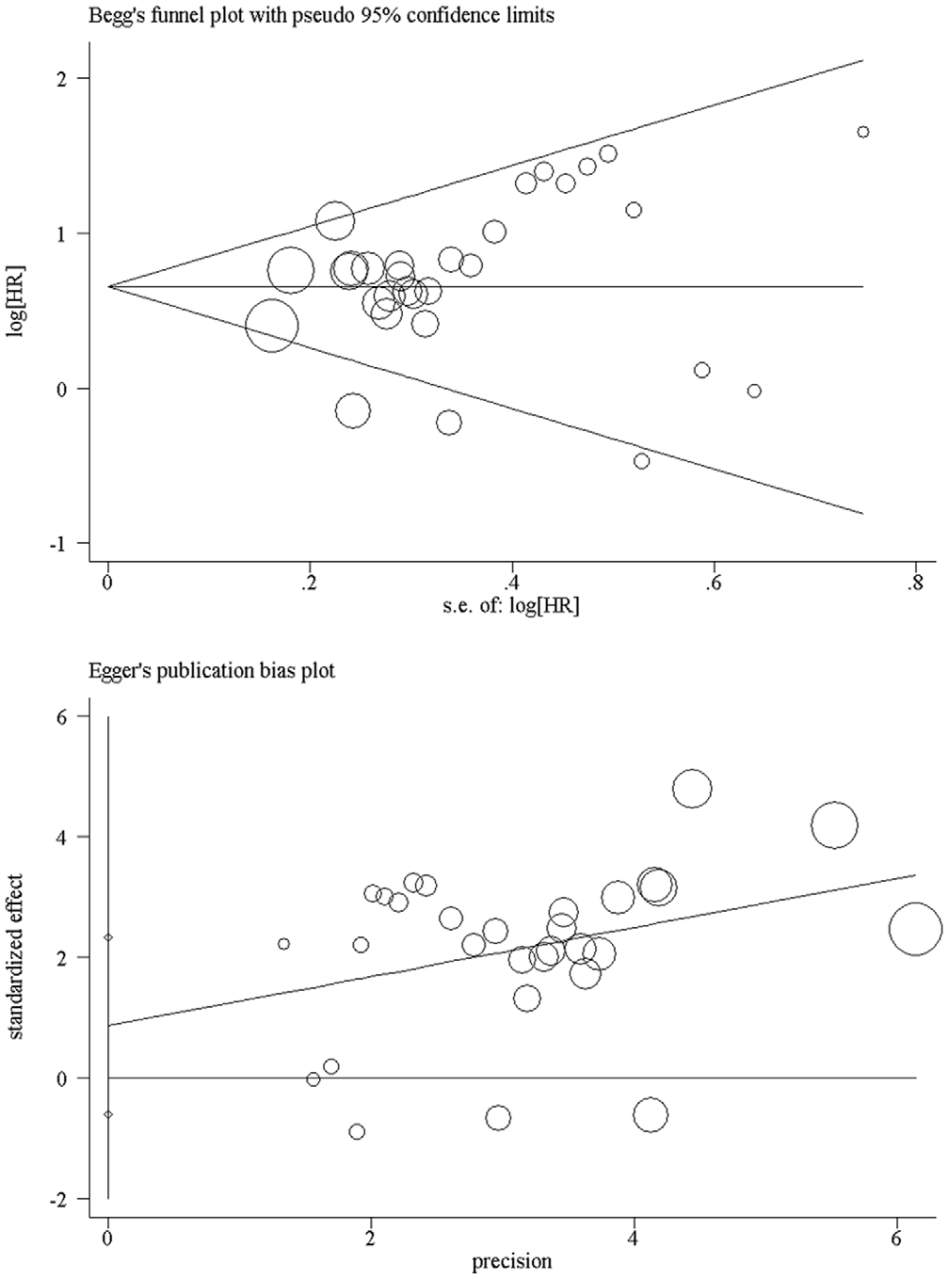
Funnel plots of Begg's and Egger's were used to detect publication bias on overall estimate. Studies are distributed symmetrically above and below the horizontal line, and suggest that the meta-analysis is absence of publication bias.

The Egger's test and Begg's funnel plot were applied for detecting publication bias in the meta-analysis. In all included studies, no funnel plot asymmetry was found, with p = 0.24 in the Egger's test (Figure 3). So, there is no evidence of publication bias detected.

## Discussion

Survivin, as a biomarker of prognosis in malignancies, has generated much interest. But the conclusions for its prognostic value are controversial. Survivin expression is an unfavorable prognostic indicator in esophageal, hepatocellular, and ovarian cancers, cholangiocarcinoma, and endometrial cancers. In contrast, favorable outcome associated with nuclear survivin has been reported for gastric, bladder, and breast cancers, ependymoma, osteosarcoma and pancreatic ductal adenocarcinoma [43], [44]. The differences in the prognostic value of survivin for NSCLC patients were also observed. Although one meta-analysis had been reported on the prognostic value of survivin in NSCLC previously [45], the authors ignored five [30], [32], [34], [37], [41] valuable studies at the time of literature searching. Furthermore, the effect size was evaluated by relative risk (RR), which measured only the number of events and took no account of when they occur are appropriate for measuring dichotomous outcomes, but less appropriate for analyzing time-to-event outcomes [46]. With a larger sample size acquired and appropriate method to aggregate the individual data, a meta-analysis to veritably evaluate the role of survivin in the prognosis of non-small cell lung cancer was performed.

The level of evidence provided by retrospective studies regarding prognostic indicators is lower than provided by randomized controlled trials. Our study used data from published trials rather than individual patient data. Although no evidence for significant heterogeneity was found, it is possible that the results of the meta-analysis could have been influenced by differences between the 28 studies. Studies may have differed with regard to the baseline characteristics of the patients included (age, histological type, differentiation or disease stage), the adjuvant treatment they might have received, the duration of follow-up and adjustments for other cofactors. Therefore, we attempted to perform a stratified subgroup analysis. As a result, the meta-analysis based on 31 literatures shows that the expression of the survivin protein is a poor prognostic factor for the survival of NSCLC who underwent surgical resection. However, when stratified analysis was conducted about different stages of NSCLC, the association was also found in stage III–IV, but not stage I–II, indicating that survivin could probably predict worse prognosis in advanced-stage NSCLC. In particular, an important issue that we need to take into account is the type of adjuvant therapy that each patient received after resection because chemotherapy and/or therapies that target the epidermal growth factor receptor, such as gefitinib or erlotinib, can change the outcome for NSCLC patients [47]. Thus, different therapies especially targeted therapies may influence the survival of NSCLC. However, the majority of published studies lacked detail regarding patient treatment. All these sources of variability could produce additional inconsistencies and cause potential selection bias. Therefore, our results need to be substantiated by further prospective studies.

We also performed a methodological assessment of the studies to avoid some selection biases (more detailed reports of significant trials) according to ELCWP scale. The absence of a detectable difference in quality score between significant and non-significant studies encourages us to perform a quantitative aggregation of the results of the individual trials. But this meta-analysis had to deal with heterogeneity problems. There was a highly significant heterogeneity among the 31 evaluable studies. Meta-regression analysis according to the type of patients, the disease characteristics, and the diversity in the techniques used to identify survivin status detected 3 studies accounting for the heterogeneity.

The heterogeneity could be explained by the fact that the technique of detecting survivin is not comparable among the studies. Most studies (77.4%) in the meta-analysis used IHC staining to study expressions of survivin. Although IHC staining is simple and cost-effective to perform, results are highly dependent on a variety of methodological factors, such as storage time, fixation method of paraffin-embedded tissues, different primary antibodies, the revelation protocols and different levels of positive (0, 10, 50%, different scores combining intensity and percentage, intensity only) [48]. Traditional survival analysis techniques (Kaplan–Meier, log-rank test) rely on variable dichotomization into high or low values or splits into multiple bins. Immunostaining cutoff point were arbitrarily selected and varied between studies. So, variability in protein expression assessment must be considered a potential source of bias. Only three studies [12], [15], [20] identified survivin using tissue microarray highlighted the importance of this high-throughput methodology allowing appropriate tissue resource rationing but also improving IHC standardization and reproducibility in large cohorts. In addition, the use of the recently published REMARK guidelines for reporting of prognostic factor studies will aid in a more complete and transparent reporting [49], thereby also increasing the number of high-quality studies that can be included in a meta-analysis.

Finally, the pooled HRs calculated in our meta-analysis may be overestimated due to publication and reporting bias. We attempted to minimize publication bias by performing the literature search as complete as possible, using PubMed, EMBASE and Chinese Biomedicine databases. However, we did not take unpublished papers and abstracts into account because the required data was unavailable. Positive results tend to be more acceptable by journals, whereas negative results often are rejected or are not even submitted for review. Of the studies investigating survivin expression in patients with NSCLC, 4 were not included in the meta-analysis due a lack of available, or calculated, survival statistics. Another potential source of bias is related to the method used to extrapolate the HR. If the HR was not reported in a study, it was calculated from the data included in the article or extrapolated from the survival curves. In fact, the method of extrapolating HR from survival curves did seem to be less reliable than when it was obtained from published statistics because this strategy did not completely eliminate inaccuracy in the extracted survival rates. In addition, each study adjusted for different covariates and only the studies that found significant results in univariate analysis performed multivariate analysis, thus pooling of results may produce bias. Language also introduces bias, and positive results tend to be published in English-language journals. Although our search was conducted without language restriction, only one study written in a Japanese language was included except for Chinese and English articles in the meta-analysis [26]. Nevertheless, no publication bias was detected using Egger's test, suggesting that the statistics obtained approximate the actual results.

In conclusion, survivin expression was associated with a poor prognosis in patients with NSCLC in the present systematic review with meta-analysis. Interestingly, our meta-analysis suggests that survivin has a detrimental effect on survival in stage III–IV NSCLC. Survivin expression as a prognostic tool at the advanced stage of NSCLC may help clinicians to make difficult therapeutic decisions. Our conclusions need to be confirmed by an adequately designed prospective study and the exact role of survivin expression needs to be determined by an appropriate multivariate analysis taking into account the classical well-defined prognostic factors for lung cancer.

## Materials and Methods

### Search strategy and selection criteria

Studies were identified via an electronic search of PubMed, EMBASE and Chinese Biomedicine Databases using the following keywords: non-small cell lung carcinoma, NSCLC, BIRC5, baculoviral inhibitor of apoptosis repeat-containing 5, survivin, prognostic, prognosis and survival. The search ended on June 2011. The meta-analysis gathered complete databases from published studies dealing with the prognostic value of survivin in patients with NSCLC who underwent surgical resection of tumor. No language of published papers was restricted. To be eligible for inclusion, studies had to meet the following criteria: (i)they measured survivin expression in NSCLC with immunohistochemistry (IHC), reverse transcription- polymerase chain reaction (RT-PCR) or fluorescence in situ hybridization (FISH); (ii) compared of overall survival between different expressions of survivin in NSCLC; (iii) hazard ratios (HRs) for overall survival according to survivin status either had to be reported or could be computed from the data presented; (iv) when the same author or group reported results obtained from the same patient population in more than one article, the most recent report or the most informative one was included. We also used a manual reference search for relevant articles, including original articles and reviews, to identify additional studies. Abstracts were excluded because of insufficient data for use. To avoid duplication of data, we carefully noted the author names and the different research centers involved.

### Data extraction

Data were extracted independently by two investigators (Zhang L.Q. and Jiang F.) by means of a predefined form. Topics in this form were first author's surname, year of publication, patient race, number of patients, histological type, disease stage, assay method, scoring protocol used, positive ratio, and survival data. In addition, discrepancies were resolved by a meeting called by Xu L.

### Assessment of study quality

Study quality was assessed independently by two investigators (Zhang L.Q. and Xu L.) by means of reading and scoring each study according to the European Lung Cancer Working Party (ELCWP) scale established by Steels et al [50]. Briefly, each item of the score was assessed using an ordinal scale (possible values 2, 1, and 0). The overall score assessed several dimensions of methodology, grouped into four main categories: (i) the scientific design; (ii) the description of the methods used to identify the abnormal of survivin; (iii) the generallizability of the results; and (iv) the analysis of the study data. Each category had a maximal score of 10 points with an overall maximum theoretical score of 40 points. When an item was not applicable to a study, its value was not taken into account in the total for the category. The final scores were expressed as percentages, with higher values reflecting a better methodological quality.

### Definition of outcomes and comparisons

The primary outcomes were the overall survival in all population and then stratified by histological type, ethnicity, stage, test method, cutoff value, hazard ratio estimate and literature written language. The effective value of overall survival was determined by the combination of HR and 95% confidence interval (CI). If a direct report of HR and 95% CI was not available, estimated value was derived indirectly from other presented data using the methods described by Tierney et al [46]. Two independent persons read the curves using Engauge Digitizer version 2.11 (free software downloaded from http://digitizer.sourceforge.net) to reduce inaccuracy in the extracted survival rates.

### Statistical analysis

The correlation between two continuous variables was measured by the Spearman rank correlation coefficient. Non-parametric tests were used to compare the distribution of quality scores according to the value of a discrete variable.

The combination of the estimated risk was obtained by calculating the log (hazard ratio) and its variance estimates. A combined HR>1 implied a worse survival for the group with survivin overexpression. This pejorative impact of survivin on survival was considered as statistically significant if the 95% CI for the combined HR did not overlap 1. To assess heterogeneity among the studies, we used the Cochran Q and I^2^ statistics: for the Q statistic, a P value<0.10 was considered statistically significant for heterogeneity [51]; for I^2^, a value>50% is considered a measure of severe heterogeneity [52], then the random-effects model was calculated according to the DerSimonian–Laird method [53]. Otherwise, the fixed-effects model (Mantel–Haenszel method) was used. The assessment of sources of heterogeneity was undertaken by meta-regression analysis [54]. One-way sensitivity analysis was performed to assess the stability of the results, namely, a single study in the meta-analysis was deleted each time to reflect the influence of the individual data set to the pooled HR [55]. A funnel plot and Egger's linear regression test were used to investigate any possible publication bias [56]. For all analyses, a two-sided P value less than 0.05 was considered to be statistically significant. All analyses were performed using STATA version 11.0 software (Stata Corporation, College Station, TX).
